# Our State Organization

**Published:** 1909-04

**Authors:** 


### OUR STATE ORGANIZATION.

A PLEA FOR A BETTER UNDERSTANDING—CHANGES THAT SHOULD BE MADE IN THE INTEREST OF HARMONY.

*To the Privates in the Rear Rank and to the Bosses—Greeting:*

In the zeal and enthusiasm attending the reorganization of our State Medical Association undue haste was made and many defects were overlooked, which under practical working (now five years) have become apparent; and these defects, together with neglect to put in operation certain provisions of the constitution and by-laws, to be presently pointed out, in a measure may account for the fact, stated in the January number of the Association's Journal (page 216), that at the end of five years there are 4050 non-members of either County or State Associations. Of this 4050 the editor says there are over 3600 regulars (and approximately 380 of minor schools, so called), while there are only 3322 members of the State Association. It will be seen that this is less than one-half of the white regulars of the State.

There are other reasons why one-half of the regular profession are holding aloof and do not come into the fold, reasons which I will point out in the course of this article.

The first defect that I call attention to is in the opening clause of the Constitution, Article II, Purposes of the Organization:

"The purposes of this Association shall be to federate and bring into one compact organization the entire medical profession of the State of Texas."

Now, the "entire medical profession of the State" includes all the sectarians (as well as the regular medical profession), the negroes and the quacks, the reputable and the disreputable. Lafayette is a licensed, registered "regular" physician. The negroes, as a rule, are licensed, registered, regular physicians.

I cannot conceive that it was ever contemplated to affiliate with homeopaths, osteopaths, eclectics or any other of the sectarians, so long as they adhere to their sectarian title, *caeteris paribus;* nor with negroes, regular or irregular, under any circumstances; nor with quacks, however "regular," irregular or defective. Ours is a semi-social, semi-scientific association, and it would be clearly impossible, even should there be a disposition to do so, for the body to assimilate this heterogeneous element. I shall offer an amendment to the Constitution to strike out this sentence and insert: "It shall be the purposes of this Association to bring into one compact organization all of the reputable white physicians of the State; and no one calling himself by any sectarian title shall be eligible to membership. Nor shall any person of the Negro, Chinese, Japanese or Mexican race be admitted." * * *

The leopard cannot change its spots. A great absurdity occurs in our By-Laws. In Section 6, in defining the duties of County Societies, it is stipulated that "It [the House of Delegates] shall especially and systematically endeavor to promote friendly intercourse between physicians of the same locality; and *shall continue these efforts until every physician* [sic] in every county in the State, *who can be made reputable* has been brought under Medical Society influence." [Italics mine.]

The process by which a rotten egg may be made good, by which a black man may be made white, or a quack varnished is not set forth; nor is it clear what standard of reputability is to guide, what constitutes reputability; nor is it made the duty of any one or set of men to do the stunt of making a man "reputable."

General meetings (seldom held) are provided for in Article VII of the Constitution; two general meetings each day.

This may be excused, perhaps, for want of time, but nothing can excuse the trick by which the majority of the members are deprived of a voice in anything. I have had much to say about this disregard of the plain letter of the Constitution, and disfranchising the members, and I here, again, and now, protest against a repetition of it. The Constitution provides for a

REFERENDUM [Art. X] in these words: "The general meeting of the Association may by a two-thirds vote of the members present order a general referendum upon any question pending before the House of Delegates, and the House of Delegates may, by a similar vote of its own members, or, after a like vote of the general meeting, submit any such question to the membership of the Association for a final vote, and if the persons voting shall comprise a majority of all the members [present?] a majority of such vote shall determine the question, and be binding upon the House of Delegates."

Again: Chapter III of By-Laws: "The general meetings shall include all registered members, delegates and guests, who shall have equal rights to participate in the proceedings and discussions, and, except guests, *to vote on pending questions.*"

It will be noted that the wording of the referendum is "may," and not "shall;" permissible, but not mandatory. It should be mandatory and I shall offer an amendment to make it so. The members should no longer be led or driven. They have a right to a voice and I shall stand for that right, as I have stood for twenty-five years for what I earnestly conceive to be the rights, interest and honor of the regular profession of legitimate, scientific medicine. The above is the loophole through which the clique in control escaped when they cut out the yearly volume of Transactions, against the will, wishes and desires of the great mass of the membership. It was an outrage not yet condoned.

This brings me to a subject which I approach with some hesitation, in the sure conviction that I will be misunderstood and falsely accused of interested motives. But I have never feared to do what I conceive to be a duty; and this is one—sure. I mean the policy and management of the Association's Journal. That it was understood by the rank and file to be intended to take the place of the Transactions goes without saying; to be a record of the doings of the organized profession, its papers, debates, acts, etc., with such records as are necessary to perpetuate the history of the Society, and that in its editorial policy it would be conciliatory, at least fair; that in its editorial columns there would be discussed new remedies, new uses for old remedies, methods of diagnosis, treatment, news of the profession, etc., etc., and I can not conceive that it was ever in the mind of even the most radical of those who put up the job on the membership that it should become the reverse of all this. I cannot conceive that it was ever dreamed that it would become a political organ of persecution, intolerance, injustice, denunciation, intimidation and bull-dozing that would antagonize a large element by denouncing as "unclean, infectious and unworthy of support" the three medical journals of the State whose editors and owners are men of the highest character and standing, old members and staunch supporters of the Association, and whose publications were always devoted to the interest of the profession and at the service of the Society; journals that did much to aid in the reform and the upbuilding of the organization, forsooth, *because* they did not, at once, fall in with the policy of the apostate homeopath and late quack of Medical Institute and Water Cure notoriety; a policy as offensive as it was high-handed; echoed by the State Association journals, accompanied by denunciation of all who chose to disregard the dictum of the "Council" as to what they shall and shall not prescribe. Those gentlemen have a large and loyal following, and the publications they control enjoy a large patronage. Their friends resent, as they do, having had this gratuitous and libelous insult thrown in their teeth. It was the newly imported editor of the *Texas State Journal of Med-*

*icine* who cast this first stone, himself not without sin; cast it unprovoked, unwarranted by any fact, because, as I said, the journals did not see fit to destroy their business by limiting their advertising patronage to proprietaries which had been censored by the immaculate, self-appointed and self-perpetuated ouncil of Pharmacy. It is paradoxical and ridiculous that at the very time this sweeping and insulting denunciation was made, the *State Journal of Medicine* was carrying certain of the "outlawed" ads, under contract; and according to the admissions in the editorial columns of the *Journal* (January, 1909), the last was only gotten rid of at the end of 1908. I cannot conceive that it was ever intended or expected that the editor of our State Association journal should be a pocket edition of the detested Simmons, and the protagonist for, champion and defender of his offensive policies, including homologation with all the sects—"unification"—and it is a matter of surprise that the Trustees would for a moment countenance or tolerate such a course. I protest against it. The *Journal* should be conducted legitimately, courteously, justly, conciliatory in tone, or abolished. While it is not to be expected that the editor would be a Crichton in accomplishment, or an Addison in style, it is demanded by every consideration that he be not insulting and unjust; and that he shall not attack with false statements and insinuations those who make war on Simmons. In other words, he must be a gentleman and treat others as gentlemen. "The purpose of the organization is," among other things, "to promote friendly intercourse among physicians." Such policy defeats this fundamental purpose.

A more conciliatory and sensible tone in the conduct of the editorial department will do much to allay the irritation engendered by the several bad breaks this party has made; and by living up to the Constitution.

"A soft answer turneth away wrath, and angry words stir up strife." The Association's editor gave the first offense; cast the first stone. I have always tried to follow the advice Polonius gave Laertes: "Beware of entering into a quarrel. But being in, so bear yourself that your adversary may beware of you." I am not of those who turn the left cheek when the right is smitten; nor would I "throw in" my vest if my coat be taken. It is my nature to hit back, and to hit hard. And I have hit hard, and when or *if* I am attacked again, it may be depended that I will hit again and hit harder still!

Now, this is all wrong. Let what I have said be pondered and acted on. Let the Trustees call a halt in the misdirection of editorial energies, and let us try to build up a white man's State Association of ethical gentlemen of the regular profession only. It is the part of wisdom. The editor of the Association's Journal has made an excellent secretary; none better; but he has a totally erroneous conception of his duties as editor. Bossism, even on a small scale, will not be tolerated. We must perfect our organization and keep it up. F. E. DANIEL.

---

THE *Texas State Journal of Medicine* says: "In this age, we should not be, as Rienzi said of his fellow Romans: 'Slaves to a horde of petty tyrants,' like envy, jealousy and personal animosities. Then, 'to be a Roman was greater than to be a king,' and in the twentieth century it is more honorable to be a high-class, broad-gauged American physician than to be a king."

The editor never spoke a truer word. For these very reasons we are protesting against the high-handed outrageous dictation of that "horde of petty tyrants" in control of the A. M. A.—Water-Cure Simmons and his "Council," who dare to dictate to the physicians of America what they shall or shall not prescribe or advertise. And we apprehend that a "high-class, broad-gauged American [Texas] physician" will not consent to affiliate socially with negroes, or even "scientifically" with irregulars. There are negroes in the A. M. A. We don't want any of it "in ours."

---

THE GOVERNOR VETOED the Tuberculosis Sanitarium bill. His objections are that "Section 12 provides that applicants for admission to the sanitarium shall have been bona fide residents of this State for two years next preceding the filing of their application. I have carefully considered this measure, and believe that the large expenditures contemplated with facilities for only 200 patients, taken from the thousands now suffering from this dreaded disease in our State, demostrates the impracticability of dealing with this important question in the manner proposed and outlined by this bill, and, it is, therefore, disapproved. The taking of so small a number into a sanitarium from the great number of sufferers from tuberculosis in this State would not meet the situation, and would hardly justify the heavy expenditure required at this time and incident to the maintenance of such institution hereafter."

---

THE BOARD OF HEALTH BILL still hangs by a slender thread of hope. It passed the Senate greatly changed, and is now in the

House, with only five days of the present session remaining (April 6th). It will be passed or killed by the time this is read. If not reached, there is yet some hope that it may get before the second called session—April 10th to May 10th. There appears to be an inclination on the part of the Legislature to enact some kind of protective law, but this particular bill seems to be objectionable, for some reason, in many respects. April 9th.—Ralston's substitute for State Board of Health bill passed the House. It gives us all essentials. Its fate now depends on joint conference committee.

---

Death of Dr. M. A. Taylor.—Dr. M. A. Taylor, the veteran physician of Austin, died at his home March 18 (ult.), aged 83 years and four months. In our December number we announced his election as President of the Seventh District Medical Association, gave a brief biographical sketch and published his picture in connection therewith, and an account of the banquet in his honor, following the meeting. Dr. Taylor was born in Ohio, November 12, 1825, graduated from Starling Medical College in 1852, removed to Austin that year and was engaged in active practice nearly fifty-six years. He was foremost in all public enterprises and in church work, and greatly beloved by all classes. Thus passes one of Austin's benefactors and shining lights in medicine.

---

